# *Bifidobacterium adolescentis* Alleviates Liver Steatosis and Steatohepatitis by Increasing Fibroblast Growth Factor 21 Sensitivity

**DOI:** 10.3389/fendo.2021.773340

**Published:** 2021-12-30

**Authors:** Xiaoxue Long, Dan Liu, Qiongmei Gao, Jiacheng Ni, Lingling Qian, Yueqiong Ni, Qichen Fang, Weiping Jia, Huating Li

**Affiliations:** ^1^ Department of Endocrinology and Metabolism, Shanghai Jiao Tong University Affiliated Sixth People’s Hospital, Shanghai Diabetes Institute, Shanghai Clinical Center for Diabetes, Shanghai, China; ^2^ Department of Medicine, Shanghai Jiao Tong University School of Medicine, Shanghai, China; ^3^ Systems Biology and Bioinformatics Unit, Leibniz Institute for Natural Product Research and Infection Biology-Hans Knöll Institute, Jena, Germany

**Keywords:** NAFLD, NASH, *B. adolescentis*, FGF21, FGF21 sensitivity

## Abstract

The gut microbiota is a newly identified contributor to the development of non-alcoholic fatty liver disease (NAFLD). Previous studies of *Bifidobacterium adolescentis* (*B. adolescentis*), a species of *Bifidobacterium* that is common in the human intestinal tract, have demonstrated that it can alleviate liver steatosis and steatohepatitis. Fibroblast growth factor 21 (FGF21) has long been considered as a biomarker of NAFLD, and recent studies have shown the protective effect of FGF21 analogs on NAFLD. We wondered whether *B. adolescentis* treatment would alleviate NAFLD via the interaction with FGF21. To this end, male C57BL/6J mice on a choline-deficient high-fat diet (CDHFD) were treated with drinking water supplemented with *B. adolescentis* for 8 weeks, followed by the acute administration of recombinant mouse FGF21 protein (rmFGF21) to conduct the FGF21 response test. Consistent with previous studies, *B. adolescentis* supplementation reversed the CDHFD-induced liver steatosis and steatohepatitis. This was evaluated on the NAFLD activity score (NAS), reduced liver enzymes, and lipid accumulation. Further studies demonstrated that *B. adolescentis* supplementation preserved the gut barrier, reduced the gut microbiota-derived lipopolysaccharide (LPS), and inhibited the hepatic TLR4/NF-κB pathway. This was accompanied by the elevated expressions of the receptors of FGF21, fibroblast growth factor receptor 1 (FGFR1) and β-klotho (KLB), in the liver and the decreased expression of FGF21. The results of FGF21 response test showed that *B. adolescentis* supplementation alleviated the CDHFD-induced FGF21 resistance. In vivo experiments suggested that LPS could suppress the expression of FGF21 and KLB in a dose-dependent manner. Collectively, this study showed that *B. adolescentis* supplementation could alleviate NAFLD by increasing FGF21 sensitivity.

## Introduction

Non-alcoholic fatty liver disease (NAFLD) has emerged as an escalating public health problem, with about 1/4 of the world’s population being affected (1), resulting in increased risks of not only liver damage but also extrahepatic diseases (2, 3). The high prevalence has aroused the curiosity of many researchers, but the mechanism of which is still not fully understood. The role of the gut microbiota in the occurrence and development of NAFLD pathogenesis and development has been demonstrated in both animal and human studies. People with NAFLD show different gut microbiota signatures compared to those of healthy people (4–6). Moreover, germfree mice on high-fat diet exhibit much less lipid accumulation in liver than control mice, suggesting the indispensable role of the gut microbiota in this disease (7). Fecal transplantation also confirmed that hepatic steatosis could be passed on from donors to germfree mice by the gut microbiota (6). Due to the important role of the gut microbiota in the development of NAFLD, multiple interventions targeting on the gut microbiota have been studied in NAFLD. Prebiotics, antibiotics, and probiotics have been shown to reduce NAFLD development (8). *Bifidobacterium adolescentis* (*B. adolescentis*), one of the common microbiota colonizing human intestinal, has shown its protective effects against the diet-induced hepatic steatosis and nonalcoholic steatohepatitis (NASH) (9, 10). The mechanism by which the gut microbiota act on NAFLD is thought to involve bile acid regulation, short chain fatty acid release, gut barrier preservation, and gut-derived toxin reduction. However, the underlying mechanisms have not been fully elucidated.

Due to the complexity of NAFLD, there is still no recommended medication for the treatment of this disease. At present, the most effective treatment for NAFLD is weight loss. Fibroblast growth factor 21 (FGF21) is an endocrine cytokine belonging to the FGF19 superfamily and is primarily produced by the liver. It serves as a regulator of many processes, such as energy homeostasis, glucose and lipid metabolism, and insulin sensitivity (11). Physiologically, FGF21 can be induced by a variety of cellular stressors and is thus characterized as a stress-responsive cytokine. Unlike most of the other FGF family members, FGF21 has a poor affinity for heparin-binding domain sulfate, which enables it to act in an endocrine way. At the same time, due to the poor affinity for heparin sulfate, FGF21 could not directly bind to the classic FGF receptors. The formation of a complex with FGF receptor and β-Klotho (KLB) is indispensable for FGF21 to exert its physiological effect (12). FGF receptor 1 (FGFR1) is the main receptor of FGF21, which is wildly distributed. But the expression of the co-receptor KLB is limited to the liver, adipose tissues, pancreases, and possibly muscles, which restrict the target tissues of FGF21 (13). As a regulator of lipid metabolism, FGF21 is closely related to NAFLD. It has been regarded as a biomarker of NAFLD since its circulating level are significantly higher in patients with NAFLD (14–16). Moreover, several clinical trials have shown that FGF21 analogs might be a promising therapeutic strategy for NAFLD. A phase IIa clinical study from The Lancet showed the effectiveness of pegbelfermin, a PEGylated human FGF21, in the treatment of NASH. Specifically, liver fat fraction, serum aminotransferases, and biomarkers of fibrosis were significantly reduced after of daily or weekly injection of pegbelfermin injections (17).

Considering the protective effect of FGF21 on NAFLD, and the fact that the liver is the first target of gut-derived metabolites and toxins, we wonder if the treatment with probiotics would alleviate NAFLD by the interaction with FGF21. Herein, we treated a NAFLD mice model with *B. adolescentis* with the attempt to test whether there is any crosstalk between *B. adolescentis* and the hepatic FGF21 pathway.

## Materials and Methods

### Animals and Treatments

8-week-old male C57BL/6J mice purchased from the GemPharmatech Company (Nanjing, China) were housed in a 12-h light-dark cycle in specific pathogen-free (SPF) facilities. The mice were subjected to normal chow diet (Research Diets, containing 10% of total calories from fat) or choline-deficient high fat diet (CDHFD; Research Diets, containing 45% of total calories from fat) and water for 16 weeks. From the start of the 17^th^ week, the mice were divided into four groups as follows: a normal chow diet control group (Control), choline-deficient high fat diet group (CDHFD-water), CDHFD + live *B. adolescentis* group (CDHFD-B.a), and CDHFD + heat-killed *B. adolescentis* group (CDHFD-hk B.a). Drinking water supplemented with *B. adolescentis* (DSMZ 20086) was administered to mice in the CDHFD-B.a group, and the same amount of heat-killed *B. adolescentis* (121°C under 225-kPa pressure for 15 min) was added in water administered to mice in the CDHFD-hk B.a group. According to the preliminary experiments, effective dose of *B. adolescentis* was 4×10^10^ CFU/day and mice would drink 8 ml water per day. The concentration of *B. adolescentis* in drinking bottles was 5×10^9^CFU/mL to ensure a dose of 4×10^10^ CFU/day to each mouse. Bottles were changed once a day to assure the activity of bacteria as reported (10). Water was supplied to mice in bottles with leak prevention to accurately measure the consumption of water and bacteria. The treatment lasted for 8 weeks. At the end of the treatment period, all mice were sacrificed after 6 h of fasting. Heart blood and tissue samples were obtained. Part of the tissues were fixed for histological analysis, and the remaining tissues were immediately snap-frozen and stored at -80°C for further investigations. All animal experimental protocols were approved by the Animal Ethics Committee of the Shanghai Jiao Tong University Affiliated Sixth People’s Hospital.

### Plasma and Tissue Analysis

Blood samples were allowed to stand for 1 h at room temperature and centrifuged at 4000 rpm for 20 min to separate the upper serum. Serum lipopolysaccharide (LPS) was measured by the Limulus Amebocyte Lysate assay (Hycult Biotech). Serum levels of triglyceride (TG), alanine aminotransferase (ALT), and aspartate aminotransferase (AST) were measured by commercial assay kits (Nanjing Jiancheng). All measurements were performed in accordance with the manufacturer’s instructions.

Hepatic TGs were quantified using Folch extraction (18). Liver samples from mice were sectioned to chloroform-methanol (2:1) to extract lipids. The organic extract was then dried and reconstituted in isopropanol. The hepatic TG levels were determined using the assay used for determining the serum TG levels. The concentration of hepatic FGF21 was measured using a Mouse FGF-21 ELISA Kit (Immunodiagnostics Limited). All measurements were performed in accordance with the manufacturer’s instructions.

### Histological Examination

Liver and ileum tissues were acquired when mice were sacrificed and using 4% paraformaldehyde to fix. After at least 48 h, these tissues were subsequently embedded in paraffin and then sectioned for further hematoxylin-eosin (HE) staining, Masson staining, immunofluorescent staining, or immunohistochemistry staining. For immunofluorescent staining, nonspecific protein binding was blocked by 5% normal goat serum. Ileum sections were incubated with anti–zona occuldens protein-1 (ZO-1, Abcam) or occludin (Abcam) antibodies. Then, samples were incubated for 1 hour with FITC-conjugated secondary antibodies (Life Technologies) and counterstained with 4,6-diamidino-2-phenylindole for visualization of cell nuclei. For immunohistochemistry staining, after the endogenous peroxidase activity had been inhibited by hydrogen peroxide (H_2_O_2_) for 20 min, sections were incubated overnight with anti-E. coli LPS antibody (Abcam), followed by staining with horseradish peroxidase–conjugated secondary antibody.

Three to six regions from each slide with immunofluorescent staining or immunohistochemistry staining were selected and scanned using the 10x objective by the Vectra imaging system (Perkin Elmer). The images acquired were analyzed with inForm software (v2.4.8, Akoya) for the calculation of histochemistry score (H-score).

NAFLD Activity Score (NAS) (19) of liver sections with HE staining were evaluated by two experienced pathologists independently, based on 4 semi-quantitatively histological features: steatosis (0-3), lobular inflammation (0-2), hepatocellular ballooning (0-2), and fibrosis (0-4). NAS from each pathologist was acquired by summing the steatosis, lobular inflammation, and hepatocellular ballooning scores unweighted. Final NAS of a mouse was acquired by averaging two scores from the pathologists.

### Calorie Measurement of Stool by Bomb Calorimetry

Stool was lyophilized with a Freezemobile12XL instrument (VirTis) at −77 °C. Following this, the caloric content of stool samples was measured by an isoperibol calorimeter instrument (HWR-15E, Shanghai Institute of Measurement and Testing Technology).

### *In Vivo* Gut Permeability Assays

The intestinal barrier function was measured using *in vivo* gut permeability assays as previously described (20). Mice were fasted for 6 h, then FITC-labeled dextran (DX-4000-FITC, Sigma-Aldrich) were administered by gavage with a dose of 500 mg/kg body weight. 1 h later, blood samples were collected through tail vein. The measurement of serum concentration of DX-4000-FITC was conducted by a fluorescence spectrophotometer (SpectraMax i3x, Molecular Devices) with an excitation wavelength of 485 nm and an emission wavelength of 535 nm.

### Mucosa-Adhered Microorganism Measurement

As described previously (21, 22), the following steps were performed to collect the mucosa-adhered microorganisms: the gut (from jejunum to rectum) was first separated from mice, and the feces in lumina was removed by squeezing. Then, intestine was sectioned longitudinally and the material adhered to the superficial layer was obtained by scraping the superficial layer of the with a sterile cotton swab. The content was transferred to sterile microtubes with DNA stabilizer and then stored at −80°C before DNA extraction.

### Bacteria DNA Extraction

About 1 g of stool sample was collected in sterile tubes and frozen at −80°C before further experiments. DNA from the fecal samples or mucosa samples was isolated using the PSP Spin Stool DNA Kit (STRATEC Molecular) according to the manufacturer’s instructions. A microvolume spectrophotometer (NanoDrop, Thermo Fisher Scientific Life Sciences) was used for the measurement of nucleic acids concentration.

### Cell Culture and Treatment

The hep1-6 cell line was used to measure whether LPS treatment would change the expression level of FGFR1 and KLB. The cells were cultured in DMEM medium with 10% FBS, 100 U/ml penicillin, and 100 mg/ml streptomycin at 37°C in a 5% CO_2_ humidified incubator. The cells were plated in six-well tissue culture dishes. When cultured to 70–80% confluency, cells were then treated by different concentration of LPS (0ng/mL,10ng/mL, 20ng/mL,50ng/mL, and 100ng/mL) for 12h, and harvested for the subsequent measurement of the mRNA level of FGFR1 and KLB.

### RNA Isolation and cDNA Synthesis

Total RNA was extracted with the TRIzol method using standard TRIzol RNA extraction protocol. TRIzol reagent (Thermo Fisher Scientific Life Sciences) was used to homogenized liver samples to isolated total RNA. A microvolume spectrophotometer (NanoDrop, Thermo Fisher Scientific Life Sciences) was used to determin RNA purity and concentration. RNA (1 μg) was reverse transcribed to generate cDNA using the PrimeScript RT reagent Kit (Takara) in accordance with the manufacturer’s protocol.

### Real-Time PCR Analysis

The gene transcript levels and bacterial DNA quantification were assessed using real-time polymerase chain reaction (RT-PCR). Sequence-specific primers used in the study were shown in **Table 1**. SYBR Green PCR Master Mix (Roche) was used for amplification reactions. RT-PCR were performed in a Light Cycler 480 system (Roche) following the manufacturer’s instructions. The cycle threshold (Ct) values of the target genes determined by the system were normalized to the Ct value of the endogenous control gene glyceraldehyde 3-phosphate dehydrogenase (GAPDH), and whereas those of *B. adolescentis* DNA were normalized to those of total bacterial DNA. Relative changes were calculated using the 2^−ΔΔCt^ method.

**Table 1:** Primers for RT-qPCR.

Gene	Forward primer (5’-3’)	Reverse primer (5’-3’)
GAPDH (mouse)	CTCATGACCACAGTCCATGC	CACATTGGGGGTAGGAACAC
Total bacteria	ACTCCTACGGGAGGCAGCAG	ATTACCGCGGCTGCTGG
*B. adolescentis*	ATAGTGGACGCGAGCAAGAGA	TTGAAGAGTTTGGCGAAATCG
*A. muciniphila*	CAGCACGTGAAGGTGGGGAC	CCTTGCGGTTGGCTTCAGAT
*M. schaedleri*	CGAGCGTTGTTCGGAGTGACTG	CCAGCCAGATTGCCGCCTTC
TLR4 (mouse)	AAGTTATTGTGGTGGTGTCTAG	GAGGTAGGTGTTTCTGCTAAG
CD14 (mouse)	GAAGCAGATCTGGGGCAGTT	CGCAGGGCTCCGAATAGAAT
FGF21(mouse)	GCCCAGCAGACAGAAGCCCAC	CAGCTGCAGGAGACT TTCGGGG
FGFR1(mouse)	GCCAGACAACTTGCCGTATG	ATTTCCTTGTCGGTGGTATTAACTC
β-Klotho (mouse)	TGGTTCGCCAACCCCATCCA	TGGGCCCGAAGGAAAAGGCA

A. muciniphila, Akkermansia muciniphila; B. adolescentis, Bifidobacterium adolescentis; CD14, cluster of differentiation 14; GAPDH, glyceraldehyde-3-phosphate dehydrogenase; M. schaedleri, Mucispirillum schaedleri; RT-qPCR, real-time quantitative polymerase chain reaction; TLR4, toll-like receptor 4; ZO-1, zonula occludens-1.

### Western Blotting

Liver tissue proteins were extracted using lysis buffer containing protease inhibitors (ST505, Beyotime Biotechnology) and phosphatase inhibitors (P1082, Beyotime Biotechnology). Protein was loaded onto sodium dodecyl sulfate polyacrylamide gel (SDS-PAGE) to separate by electrophoresis. After the target protein was separated, the protein would be transferred onto polyvinylidene fluoride (PVDF) membrane (Millipore) and blocking in 5% bovine serum albumin (BSA), followed by incubation with primary antibodies at 4°C overnight. On the next day, secondary antibodies would be incubated with the membrane. Immunoreactivity was detected using enhanced chemiluminescent autoradiography (Millipore). Chemiluminescence was determined using ChemiDoc Imageing System (BIO-RAD) or Optimax X-ray Film Processor (Protec GmbH & Co. KG). The Image J software (National Institutes of Health, USA) was used for band quantification. The primary antibodies used for western blotting were against Phospho-NF-κBp65 (Ser536) (3033, Cell Signaling Technology), NF-κBp65(8242, Cell Signaling Technology), KLB (AF2619, R&D), FGFR1 (12511, Cell Signaling Technology), extracellular signal−regulated protein kinase 1/2 (Erk1/2, 9102, Cell Signaling Technology), phosphorylated (p)- Erk1/2 (Thr202/Tyr204) (9101, Cell Signaling Technology), and glyceraldehyde 3-phosphate dehydrogenase (GAPDH,5174, Cell Signaling Technology).

### FGF21 Response Test

After 8-week intervention, mice were conducted theFGF21 response test (**Figure 4G**). Under a general anesthetic, left lobe liver of mice was ligated and part of it were sectioned and flash frozen with liquid nitrogen. Then 2 mg/kg recombinant mouse FGF21 (rmFGF21) or vehicle was injected to mice through inferior vena cava. 15min later, part of liver from right lobe were collected. Protein from these liver samples would be used to detect the level of Erk1/2 phosphorylation.

### Statistical Analysis

All statistical analyses were performed using GraphPad Prism (GraphPad Software). Results were presented as mean ± S.E.M. (standard error of the mean). One-way analysis of variance with the consequent *post hoc* test of Fisher’s LSD was applied. A p-values of <0.05 was considered statistically significant.

## Results

### *B. adolescentis* Supplementation Attenuated the Diet-Induced Liver Steatosis and Steatohepatitis in Mice With Diet-Induced NASH

**Figure 1:**
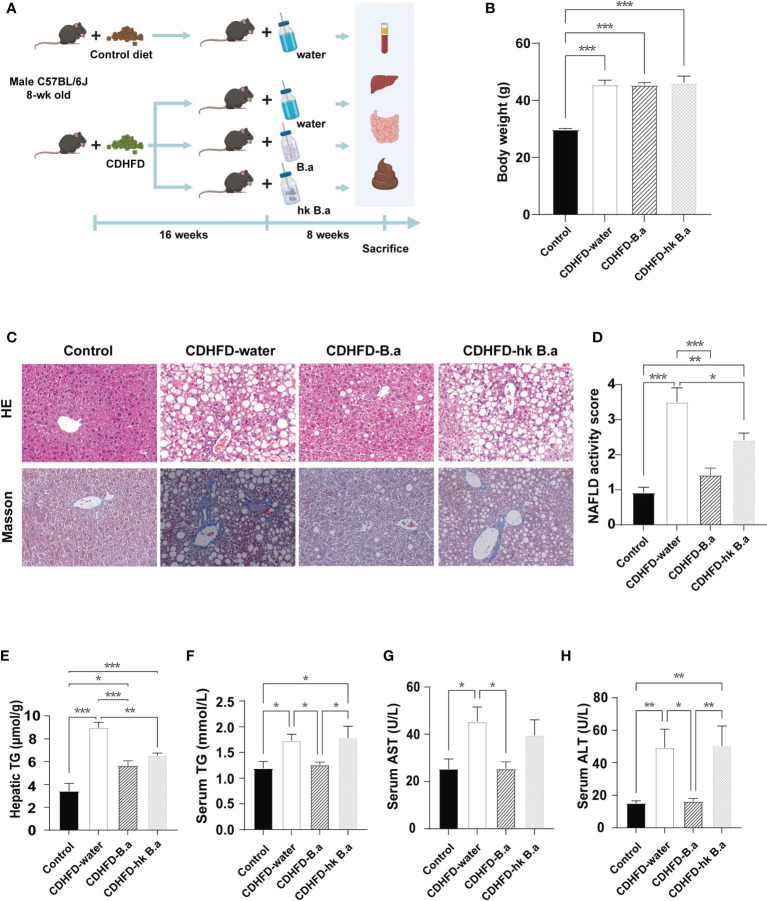
*B. adolescentis* supplementation alleviated the diet-induced liver steatosis and steatohepatitis in mice. **(A)** The overall design of the animal experiment. C57BL/6J mice on CDHFD were given drinking water (CDHFD-water), water with live *B. adolescentis* (CDHFD-B.a), or heat-killed (CDHFD-hk B.a) for 8 weeks. The experiment schematic was created using BioRender.com. **(B)** Body weight in each group after intervention (n = 6 in each group). **(C)** Histology of liver with HE and Masson staining. **(D)** NAS (n = 6 in each group). **(E)** TG level in liver (n = 6 in each group). **(F)** The level of serum TG (n = 6 in each group). **(G)** The level of serum AST (n = 6 in each group). **(H)** The levels of serum ALT (n = 6 in each group). ALT, alanine aminotransferase; AST, aspartate aminotransferase; CDHFD, choline-deficiency high fat diet. HE, hematoxylin- Eosin; NAS, NAFLD activity score; TG, triglyceride. Data are presented as mean ± SEM. Significance was determined by one-way ANOVA with Fisher’s LSD multiple-comparison analysis. *p < 0.05; **p < 0.01; ***p < 0.001.

Eight-week-old male C57BL/6J mice on control diet or CDHFD were allocated to treatment of drinking water (control; CDHFD-water), water with *B. adolescentis* (CDHFD-B.a), or water with heat-kill *B. adolescentis* for 8 weeks (**Figure 1A**), followed by further assessments. The supplementation of *B. adolescentis* in drinking water did not alter food intake nor body weight, as well as the water consumption (**Supplementary Figures 1A, B**; **Figure 1B**). And we measured the energy content in feces to investigate whether the absorption of the nutrients was affected. No significant difference was found among the four groups (**Supplementary Figure 1C**). Hepatic pathology showed that compared to control diet, CDHFD-induced massive accumulation of fat and mild fibrosis in mice (**Figures 1C, D**). Supplementation with living *B. adolescentis* attenuated these lesions and reduced the NAS (**Figures 1C, D**). In accordance with the histology findings, *B. adolescentis* abrogated the increase of circulating and hepatic levels of TGs induced by CDHFD (**Figures 1E, F**). The lower levels of AST and ALT in CDHFD-B.a group indicated a reversal of diet-induced liver damage (**Figures 1G, H**). The heat-killed *B. adolescentis* also show some benefits on NAS, hepatic TG, and serum AST. But it’s effect is weaker than live *B. adolescentis* (**Figures 1D, E, G**).

### Supplementation of *B. adolescentis* Decreased Intestinal Permeability and Lipopolysaccharide (LPS) Infiltration

**Figure 2:**
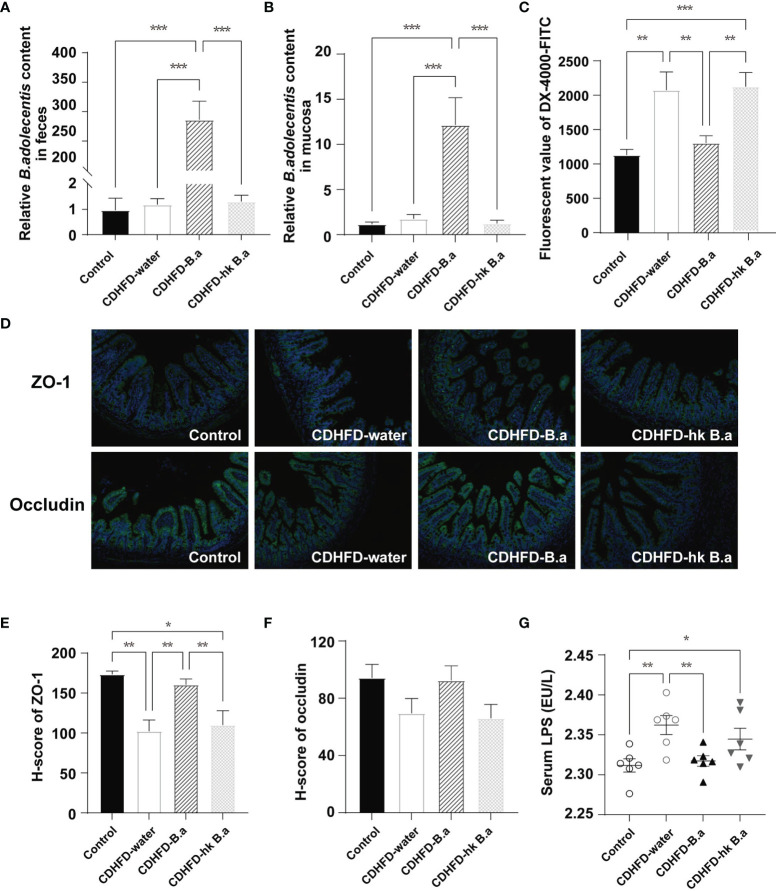
Effect of *B. adolescentis* on intestinal permeability. Mice were grouped and treated as in **Figure 1**. **(A, B)** Abundance of *B.adolescentis* in feces and mucosa after intervention determined by qPCR (n = 6 in each group). **(C)** Serum concentrations of DX-4000-FITC at 1 hour after oral gavage (n = 6 in each group). **(D)** ZO-1and occludin and in ileum detected by immunofluorescent staining. **(E, F)** H-score of ZO-1 and occludin measured in ileum section with immunofluorescent staining. **(G)** The level of serum LPS (n = 6 in each group). DX-4000-FITC, fluorescent-labeled dextran; H-score, histochemistry score; LPS, lipopolysaccharide; ZO-1, zonula occludens-1. Data are presented as mean ± SEM. Significance was determined by one-way ANOVA with Fisher’s LSD multiple-comparison analysis. *p < 0.05; **p < 0.01; ***p < 0.001.

The *B. adolescentis* content in fecal and gut mucosa after treatment was measured by the real-time PCR analysis. 4.01*10^10^ CFU/day of *B. adolescentis* intake from drinking water increased its amount both in feces and in gut mucosa (**Figures 2A, B**). Another two prominent microbes were not changed (**Supplementary Figures 1D, E**). Changes in gut permeability have been associated with NAFLD development. In particular, the increase in gut permeability lead to the entry of gut-derived metabolites into circulation, such as ethanol and LPS, which affect NAFLD development (23). To investigate whether *B. adolescentis* administration could decrease intestinal permeability, an *in vivo* gut permeability assay was performed. 1 hour after gavage of fluorescent-labeled dextran (DX-4000-FITC), its serum level in CDHFD-water group was twice as high as that in control group, indicating an increase in intestinal permeability (**Figure 2C**). Such an increase in intestinal permeability was significantly abolished by supplementation with live *B. adolescentis* (**Figure 2C**). The gut permeability was largely regulated by the tight junctions between epithelial cells of intestine, which form a physical barrier defending the intrusion of pathogens and bacterial products. Therefore, we wonder whether *B. adolescentis* could affect the level of epithelial tight junction protein ZO-1 and occludin. As immunofluorescent staining of ileum sections showed (**Figures 2D, E**), mice on CDHFD have lower ZO-1 than their counterparts on control diet, whereas those with *B. adolescentis* have similar level with control mice. The level of occludin showed the same trend as ZO-1 although not statistically different (**Figure 2F**). In line with the change in gut permeability, serum LPS concentration was higher in mice on CDHFD, whereas *B. adolescentis* treatment could decreased the diet induced elevation of LPS (**Figure 2G**).

### The LPS/Toll-Like Receptor 4 (TLR4)/Nuclear Factor-κB (NF-κB) Axis Was Suppressed by *B. adolescentis*

**Figure 3:**
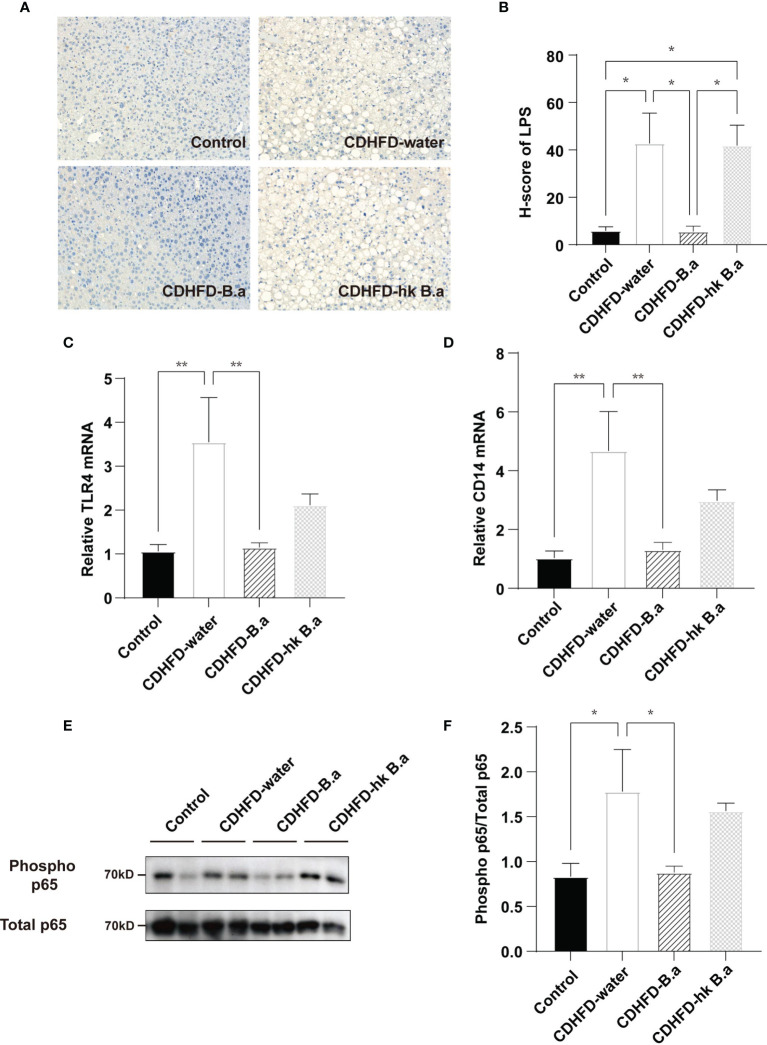
Effects of *B. adolescentis* on TLR4 pathway. Mice were grouped and treated as in **Figure 1**. **(A)** LPS location in liver detected by immunohistochemical staining. **(B)** H-score of LPS measured in ileum section with immunofluorescent staining. **(C)** The transcription of TLR4 in liver (n = 6 in each group). **(D)** The transcription of CD14 in liver (n = 6 in each group). **(E)** Phosphorylation of the p65 NF-kB subunit detected by western blot analysis. **(F)** Quantification of the relative phosphorylation of the p65 NF-kB subunit (n = 4). CD14, cluster of differentiation 14; H-score, histochemistry score; LPS, lipopolysaccharide; NF-kB, nuclear transcription factor-kappa B; TLR4, toll-like receptor 4. Data are presented as mean ± SEM. Significance was determined by one-way ANOVA with Fisher’s LSD multiple-comparison analysis. *p < 0.05; **p < 0.01.

To investigate whether the change in circulation LPS would affect liver, we measured the localization of gut-derived LPS in liver and by quantifying the H-score of LPS we discovered a similar trend with serum LPS (**Figures 3A, B**). LPS infiltration in liver has been shown to increase liver damage through a TLR4-mediated pathway (24). As the main receptor of LPS, the levels of TLR4 and CD14 were upregulated in the CDHFD-water group compared to those of control group (**Figures 3C, D**). The activation of TLR4/NF-κB signaling pathway in CDHFD mice was confirmed by the increased phosphorylation level of a NF-κB subunit p65 (**Figures 3E, F**). However, supplementation with live *B. adolescentis* abolished the CDHFD-induced upregulation of the TLR4 and CD14 (**Figures 3C, D**). And the phosphorylation level of NF-κB was also decreased by *B. adolescentis* treatment (**Figures 3E, F**). Collectively, a suppression of LPS/TLR4/NF-κB pathway was discovered in mice supplemented with *B. adolescentis*.

### *B. adolescentis* Supplementation Alleviated FGF21 Resistance

**Figure 4:**
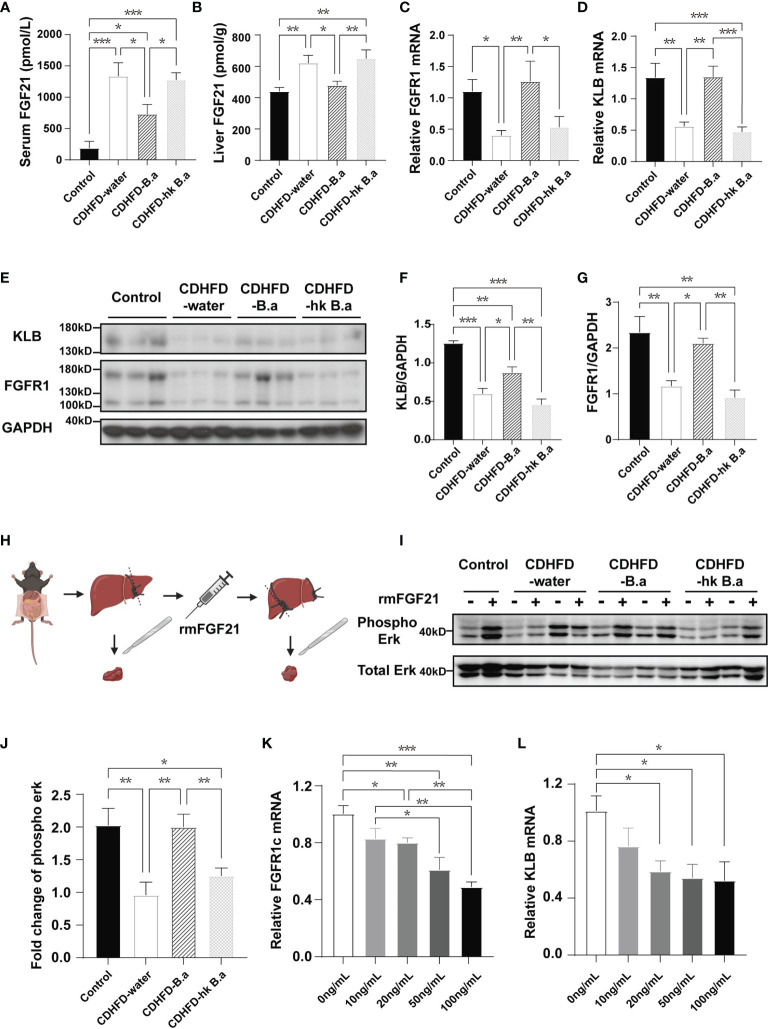
*B. adolescentis* supplementation alleviated FGF21 resistance. Mice were grouped and treated as in **Figure 1**. **(A)** The serum level of FGF21 (n = 6 in each group). **(B)** FGF21 levels in liver measured by ELISA (n = 6 in each group). **(C)** The transcription of FGFR1 in liver (n = 6 in each group). **(D)** The transcription of KLB in liver (n = 6 in each group). **(E)** The expression of FGFR1 and β-Klotho in liver detected by western blot analysis. **(F)** Quantification of the FGFR1 expression normalized to GAPDH (n = 3). **(G)** Quantification of the KLB expression normalized to GAPDH (n = 3). **(H)** Experiment schematics for FGF21 response test. The experiment schematic was created using BioRender.com. **(I)** Phosphorylation of Erk1/2 (Thr202/Tyr204) before and after rmFGF21 administration (2mg/kg) detected by western blot analysis. **(J)** Quantification analysis of the fold change of Erk1/2 phosphorylation (n = 4-6). FGF21, fibroblast growth factor; FGFR1, Fibroblast growth factor receptor 1; GAPDH, glyceraldehyde-3-phosphate dehydrogenase; KLB, β-klotho; rmFGF21, recombinant mouse fibroblast growth factor 21. **(K)** The transcription of FGFR1 in hep1-6 after the treatment with LPS. **(L)** The transcription of KLB in hep1-6 after the treatment with LPS. Data are presented as mean ± SEM. Significance was determined by one-way ANOVA with Fisher’s LSD multiple-comparison analysis. *p < 0.05; **p < 0.01; ***p < 0.001.

LPS injection has been shown to suppress FGF21 signaling in mice (25). To investigate if circulating LPS can alter the signaling of the hepatic FGF21 pathway, we examined the expression of FGF21, as well as FGFR1 and KLB in the liver. In accordance with previous findings, compared to control diet, CDHFD induced a higher expression of FGF21(**Figures 4A, B**). Despite that CDHFD increased the level of FGF21, the expression of FGF21 receptors was downregulated (**Figures 4C–G**), which indicate a potent FGF21 resistant state in these mice. By supplementation with *B. adolescentis*, the CDHFD induced FGF21 elevation was reduced (**Figures 4A, B**), accompanied by the upregulation of receptors (**Figures 4C–G**). To measure the response to FGF21 in CDHFD-B.a group, we administered recombinant mouse FGF21 (rmFGF21) or vehicle to mice (**Figure 4H**), and detected the change of Erk1/2 phosphorylation. As shown in **Supplementary Figures 1F, G**, the technique itself could not produce the downstream phosphorylation of Erk1/2. And the level of Erk1/2 phosphorylation was significantly higher in CDHFD-B.a group than CDHFD-water group or CDHFD-hk B.a group in response to FGF21 (**Figures 4I, J**). It confirmed that treatment with live *B. adolescentis* increase the liver FGF21 sensitivity in mice. To testify that *B. adolescentis* increased liver FGF21 sensitivity by reducing LPS, we treated the hep1-6 cells with different concentration of LPS. Correspondently, the expression of FGFR1 and KLB was suppressed by LPS in a dose-dependent manner (**Figures 4K, L**).

## Discussion

As the research on the gut microbiota progress, the close relationship between gut microbiota and NAFLD has been discovered. Herein, we provide an evidence that a kind of probiotics *B. adolescentis* could markedly reduce diet-induced NAFLD in mice, and that this protective effect may be related with the hepatic FGF21 pathway *via* LPS. Both exogenous and endogenous LPS have been reported to play a pivotal role in the development in NAFLD through TLR4 pathway (24, 26, 27). Consistent with our *in vitro* study, previous study has suggested that LPS injection could repress the KLB expression *in vivo* (25). By activating TLR4 pathway, LPS can induce the release of inflammatory cytokines, such as interleukin 6 (IL-6), tumour necrosis factor α (TNFα), and IL-1β. Administration of TNFα in adipose cells represses KLB expression and impairs FGF21-mediated glucose transporter 1 expression and glucose uptake (28). Furthermore, studies have shown that these three inflammatory cytokines could also repress KLB expression in a dose-dependent manner in hepatocytes (25, 29).

FGF21 resistance is mainly based on the fact that high endogenous levels of FGF21 appear to be ineffective or not as expected in metabolic regulation, but high pharmacological doses of FGF21 can induce the expected results. This was first proposed by Fisher et al. (30). They discovered that obese mice exhibited marked deficits in the ability of the peptide to initiate signals through the ras-raf-MAPK cascade in response to FGF21, accompanied by decreased expression of KLB and FGF receptors (30). The decreased FGF21 receptor complex is deemed to be an important mechanism leading to FGF21 signal impairment. KLB expression in fatty livers improves FGF21 signalling (31). However, using adipose-specific KLB transgenic mice to maintain the KLB levels in adipose tissue did not increase FGF21 sensitivity (32). More physiological and pharmacological studies are needed to elucidate the mechanisms underlying FGF21 resistance.

Although the benefit of FGF21 on NAFLD have been testified in many studier, it is still controversial whether the liver is a direct target organ for FGF21 action or indirect target *via* the adipose tissue or central nervous system (11). Expression of FGFR1c in adipose tissue is higher than liver making it the main target tissue of FGF21. However, it can also act independent of adipose (33). Studies have shown that FGF21 could inhibit mTORC1 in hepatocytes to control hepatic insulin action and maintain glucose homeostasis (34), while acute FGF21 treatment could induce hepatic expression of key regulators of gluconeogenesis, lipid metabolism, and ketogenesis (35). More recently, FGF21 was found to be dispensable in the regulation of lysosomal function, which is related to lipophagy (31, 36). Fgf21 deficiency impairs hepatic lysosomal function by blocking transcription factor EB (TFEB), a master regulator of lysosome biogenesis and autophagy (36), resulting increased lipid droplet accumulation in liver. Thus, lipophagy might be another direct mechanism by which FGF21 alleviates NAFLD. Besides, study have suggested that FGF21 could reduce the expression of TNF-α, IL-1β, IL-6 and IFN-γ and increased the level of IL-10 in a dose-dependent manner in LPS-stimulated RAW 264.7, indicating an anti-inflammatory effect (37). Apart from macrophages, FGF21 could also regulate the activation of hepatic stellate cells (38) which is important during the development of liver fibrosis. Also, treatment with FGF21 could reduce apoptosis and the level of oxidative stress during liver injury, thereby contribute to the repairment of liver function (39). The mechanism by which FGF21 alleviates NAFLD needs more studies to be further confirmed.

Unlike in the mouse intestine, *B. adolescentis* is the dominant species in the human intestine. Supplementation with *B. adolescentis* not only increase its content, could also modifies the gut microbiota (40), which contributes towards reducing LPS production in gut. Previous studies have found that *B. adolescentis* increased serum leptin concentrations and induced the expression of thermogenesis- and lipid metabolism-related genes in brown adipose tissue (40). Furthermore, *B. adolescentis* can also specifically induce intestinal Th17 cell accumulation (41, 42), which resembled the effect of FGF21 analogue on liver Th17 cells (43). Moreover, *B. adolescentis* is also a key member of the human gut microbiota in the production of GABA, highlighting its potential implication in gut-brain axis interactions (44). Collectively, supplementation with *B. adolescentis* seems to have a profound impact on the health of hosts.

Besides the supplementation with live *B. adolescentis*, the supplementation with heat killed *B. adolescentis* also showed some benefit on hepatic steatosis and inflammation, although it is weaker than live *B. adolescentis*. This indicating that some content of the bacteria could still work even though the bacteria itself was inactivated. Similarly, in previous study, another bifidobacteria (*Bifidobacterium breve* M-16V) were found to have immune-modulating effects and affected intestinal metabolism even though it had been heat-killed (45). Since heat-killed bacteria have less safety concern, it may in the future be an alternative treatment to live bacteria.

In conclusion, we have suggested a potent interaction between the gut microbiota, hepatic endocrine system, and the development of liver steatosis and steatohepatitis. Live *B. adolescentis* supplementation markedly alleviated the diet-induced NAFLD in mice and increased the sensitivity to FGF21 by suppressing the LPS/TLR4/NF-κB pathway.

## Data Availability Statement

The original contributions presented in the study are included in the article/**Supplementary Material**. Further inquiries can be directed to the corresponding authors.

## Ethics Statement

The animal study was reviewed and approved by The Animal Ethics Committee of the Shanghai Jiao Tong University Affiliated Sixth People’s Hospital.

## Author Contributions

HL and WJ designed the experiment. XL and DL carried out the experiment and wrote the manuscript with support from HL and QF. The animal experiments were conducted with the help of QG and JN. Data analysis were contributed by LQ and YN. All authors helped shape the analysis, research, and manuscript. All authors contributed to the article and approved the submitted version.

## Funding

This work was supported by Excellent Young Scholars of NSFC (82022012), General Program of NSFC (81870598), and Two Hundred Program from Shanghai Jiao Tong University School of Medicine (20191830) to HL; the National Natural Science Foundation of China (NSFC) major international (regional) joint research project (81220108006), NSFC‐NHMRC joint research grant (81561128016), Shanghai Municipal Key Clinical Specialty, and The National Key Research and Development Program of China (2018YFA0800402) to WJ.

## Conflict of Interest

The authors declare that the research was conducted in the absence of any commercial or financial relationships that could be construed as a potential conflict of interest.

The handling editor declared a past co-authorship with the authors QG, JN, LQ, YN, QF, WJ, and HL.

## Publisher’s Note

All claims expressed in this article are solely those of the authors and do not necessarily represent those of their affiliated organizations, or those of the publisher, the editors and the reviewers. Any product that may be evaluated in this article, or claim that may be made by its manufacturer, is not guaranteed or endorsed by the publisher.

## Supplementary Material

Supplementary Figure 1 Mice were grouped and treated as in **Figure 1**. **(A)** Average food intake and **(B)** water consumption of mice during the treatment period (n = 6 in each group). **(C)** The fecal caloric content in each group after intervention (n = 6 in each group). **(D, E)** Abundance of *A. muciniphila* and *M. schaedleri* in feces of four groups after intervention determined by qPCR (n = 6 in each group). **(F, G)** Phosphorylation of Erk1/2 (Thr202/Tyr204) detected by western blot analysis before and after administration of rmFGF21 (2mg/kg) or vehicle (n = 3). *A. muciniphila*, *Akkermansia muciniphila*; CDHFD, choline-deficient high-fat diet; *M. schaedleri*, *Mucispirillum schaedleri*; rmFGF21, recombinant mouse fibroblast growth factor 21; Data are presented as mean ± SEM. Significance was determined by one-way ANOVA with Fisher’s LSD multiple-comparison analysis in. *p < 0.05; **p < 0.01; ***p < 0.001.
