# Supplementary figures and images for: Bifidobacterium adolescentis Alleviates Liver Steatosis and Steatohepatitis by Increasing Fibroblast Growth Factor 21 Sensitivity
Source: Front Endocrinol (Lausanne). 2021 Dec 30;12:773340. doi: 10.3389/fendo.2021.773340 (PMC8756294; doi:10.3389/fendo.2021.773340)

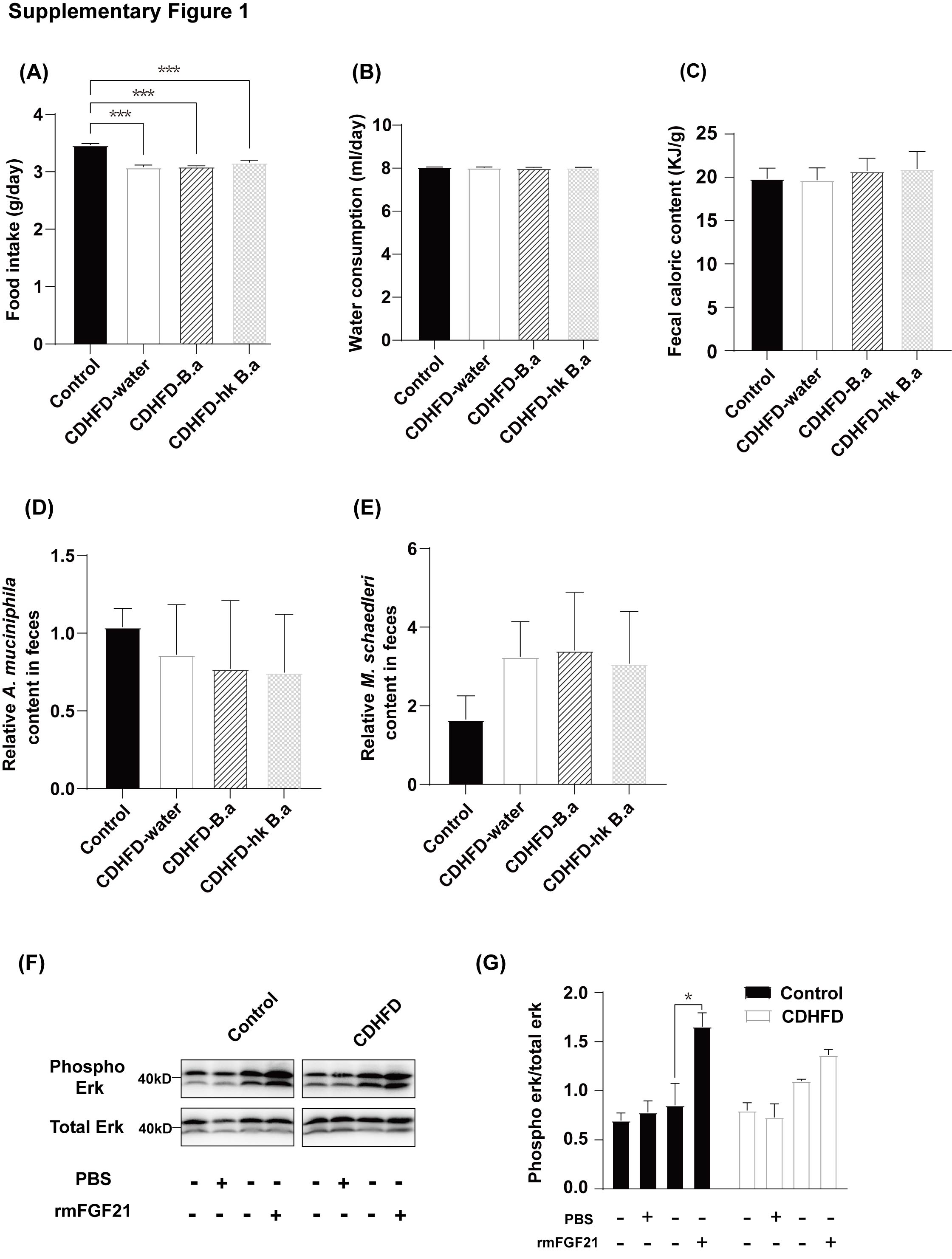

Supplement: Supplementary Figure 1 — Mice were grouped and treated as in Figure 1 . (A) Average food intake and (B) water consumption of mice during the treatment period (n = 6 in each group). (C) The fecal caloric content in each group after intervention (n = 6 in each group). (D, E) Abundance of A. muciniphila and M. schaedleri in feces of four groups after intervention determined by qPCR (n = 6 in each group). (F, G) Phosphorylation of Erk1/2 (Thr202/Tyr204) detected by western blot analysis before and after administration of rmFGF21 (2mg/kg) or vehicle (n = 3). A. muciniphila, Akkermansia muciniphila; CDHFD, choline-deficient high-fat diet; M. schaedleri, Mucispirillum schaedleri; rmFGF21, recombinant mouse fibroblast growth factor 21; Data are presented as mean ± SEM. Significance was determined by one-way ANOVA with Fisher’s LSD multiple-comparison analysis in. *p < 0.05; **p < 0.01; ***p < 0.001. [file Image_1.jpeg]
